# Patient and Caregiver Perceptions of Caregiving Contributions During Cancer Clinical Trials: A Mixed‐Methods Study

**DOI:** 10.1002/cam4.70488

**Published:** 2025-01-09

**Authors:** Janine Cerutti, Maria C. Lent, Randall F. Holcombe, Maija Reblin

**Affiliations:** ^1^ Department of Psychological Science The University of Vermont Burlington Vermont USA; ^2^ University of Vermont Cancer Center University of Vermont Burlington Vermont USA; ^3^ Larner College of Medicine University of Vermont Burlington Vermont USA

**Keywords:** cancer, caregivers, clinical trial, mixed methods, oncology, qualitative research, rural

## Abstract

**Objective:**

Caregivers play crucial roles in cancer treatment and outcomes. However, little is known regarding how caregivers support patients during cancer clinical trials. The aim of this study was to gain insight into the caregiver experience of rural and urban patients enrolled in cancer clinical trials.

**Methods:**

As part of a quality improvement study, 21 patient–caregiver dyads were interviewed using closed and open‐ended interview questions. We analyzed quantitative and qualitative data on patient and caregiver perceptions of caregiver contributions and explored differences in the reported caregiving experience between rural and urban participants.

**Results:**

While patient–caregiver dyads showed significant disagreement in the symptoms/medication management domain, with caregivers tending to acknowledge the contribution while patients did not (*χ* ^2^ (1, 21) = 5.82, *p* = 0.016), both groups generally showed agreement in their perceptions of caregiver involvement and reported similar levels of involvement across the other six assessed domains. Qualitative analysis revealed three themes: patient independence, invisible support, and accepted forms of support. Despite patients valuing independence, patients benefited from caregivers' unseen support, and providing emotional support and attending appointments were widely accepted forms of support among patients. No meaningful differences in caregiver contributions were found between rural and urban patient–caregiver dyads.

**Conclusion:**

Our study revealed that caregivers are assisting patients in often unseen and underestimated ways during cancer clinical trials, highlighting their multifaceted role. Cancer clinical trials should implement a family‐centered approach, especially for rural caregivers, to enhance patient retention and outcomes.

## 1 Background

The role of family caregivers in cancer care is increasingly recognized as the caregiver role in the healthcare system expands. The term caregiver refers to a partner, family member, or close friend who most often helps the person with cancer at home [1]. Research indicates that caregivers play a vital role in improving patient cancer outcomes and reducing overall health care costs [2, 3]. However, the responsibilities of providing care can lead to burden, particularly for caregivers who spend more time providing care, have competing responsibilities, or have their own health challenges. As such, caregiving can have negative effects on caregivers' physical and psychological well‐being [4, 5]. Poor caregiver mental and physical health can, in turn, impact patient function and quality of life [6, 7, 8].

The challenges associated with providing care are compounded for caregivers living in rural areas. In addition to being more likely to be older, have their own health challenges, and have lower socioeconomic status, rural caregivers have less access to well‐coordinated care and services can be difficult due to geographic barriers and limited resources [9, 10]. In fact, recent studies have shed light on the experiences of rural cancer caregivers, highlighting their unique and unmet social needs [11, 12, 13, 14]. Given these challenges, supporting caregivers is crucial for enhancing cancer outcomes, particularly in rural areas where needs are more pronounced.

Cancer clinical trial participation is associated with high‐quality care [15]. Unfortunately, rural patients are disproportionately less likely to have access to and to participate in clinical trials [16]. Further, little is known regarding how patient clinical trial participation affects the caregiving experience, despite a vast body of research on the patient experience of participating in cancer clinical trials [17, 18]. There are few exceptions to the limited research on caregiving during cancer clinical trials. One recent qualitative analysis explored caregivers' perceptions on the burden and benefits associated with their loved ones' cancer clinical trial participation [19]. The study found that caregivers experienced distress across a variety of domains, including financial, emotional, and psychological. Similarly, a mixed‐methods study examining patient and caregiver experiences during Phase 1 cancer clinical trials found that caregivers reported a higher level of burden and distress than patients [20]. Another recent qualitative analysis, focused on the barriers and facilitators to cancer clinical trial enrollment among older patients and their caregivers, found that access to new treatments and physician recommendations were common facilitators to enrollment, while concerns about side effects and limited information about clinical trials posed barriers [21]. Despite these findings, a gap remains in understanding how caregiving perceptions during cancer clinical trials shape the experiences of both patients and their caregivers. Understanding caregivers' roles and experiences during cancer clinical trials is crucial, given the essential role that caregivers play in cancer care.

The current study aimed to explore the experiences of caregivers of rural and urban patients enrolled in cancer clinical trials. To achieve a comprehensive understanding of these experiences, we employed a mixed‐methods approach, integrating both qualitative and quantitative data through close‐ and open‐ended interview questions. We compared data on patient and caregiver perceptions of caregiver contributions and explored differences in the reported caregiving experience between rural and urban participants.

## 2 Methods

### 2.1 Study Design

This is a secondary data analysis from a larger quality improvement study assessing barriers and facilitators to participation in cancer clinical trials. The study was determined to be exempt from approval by the University of Vermont Institutional Review Board. As part of the parent study, members of the research team developed a set of interview questions aimed at comprehensively exploring the experiences of patients and caregivers in clinical trial participation. A trained member of the quality improvement study team conducted individual telephone interviews with patients and caregivers separately. All interviews were audio‐recorded, transcribed verbatim, and anonymized before the coding process. Verbal consent was obtained for interviews, recordings, and use of anonymized responses at the start of each interview.

The interview included questions about barriers and facilitators to clinical trial participation and ways in which caregivers assisted patients (i.e., caregiver contributions) during clinical trial participation. In addition to obtaining demographic information, both patients and caregivers were asked whether they [or their caregiver] assisted in each of the following seven caregiver contribution domains: coordinating appointments, transportation to appointments, providing emotional support, treatment decision‐making, medication/symptom management, insurance/finances, and arranging/providing alternative care for children, grandchildren, or aging adults. These domains were identified and selected as common caregiving tasks during cancer treatment following a review of the literature [8].

#### 2.1.1 Participants

Potentially eligible patients were identified through the electronic medical record of the University of Vermont Medical Center and affiliated Central Vermont Medical Center. Patients were eligible for study participation if they (1) had a cancer diagnosis and (2) had newly enrolled in an active cancer treatment clinical trial between January 2022 and May 2023. Patients' caregivers were identified subjectively during patient phone interviews. The study team member conducting interviews asked each patient if there was a person, such as a spouse, family member, or friend, who might have assisted them during cancer treatment. If the patient identified such a person, the interviewer inquired whether this individual would be interested in participating in a similar interview to capture caregiver experiences during patient clinical trial participation. While the term “caregiver” was used during the interviews, the study team member noted this term may not resonate with everyone, as the relationship dynamics varied.

### 2.2 Data Analyses

#### 2.2.1 Quantitative Analyses

Descriptive analyses on participant demographics were generated. Based on household zip codes, participants were classified as living in a rural or urban location using the 2010 Rural–Urban Commuting Area (RUCA) codes [22]. Responses to close‐ended questions on the seven caregiver contribution domains were coded (1 = yes, 0 = no) and summed to create a total caregiver contribution measure. McNemar's chi‐square tests were conducted to assess the level of agreement in reported caregiver contributions between patient–caregiver dyads. All quantitative analyses were performed in R Statistical Software [23].

#### 2.2.2 Qualitative Analysis

The research team employed a directed content analysis approach [24] to analyze the interview transcriptions. First, the team met to discuss a systematic coding process and structured codebook, based on the semi‐structured interview questions. Then, each team member was randomly assigned two patient and one caregiver transcript to code for significant themes using the codebook scheme. Using the constant comparison method, the team met to discuss shared themes across codes, resolve any issues or disagreements through discussion, and create a finalized codebook. Once the codebook was finalized, all transcripts were randomly assigned and independently coded by a member of the research team. These codes were then discussed within the team to resolve conflicts, and in a synthesis review of the coding, major themes were extracted by the research team. After data analyses were completed, the research team integrated the quantitative and qualitative data through discussion and interpreted the results accordingly.

## 3 Results

### 3.1 Quantitative Results

#### 3.1.1 Participant Characteristics

In the parent study, 39 total patients were contacted, of which 30 agreed to participate in a telephone interview regarding their experiences during their cancer clinical trial. Twenty‐six of the 30 patients provided consent for the interviewer to contact their caregiver (i.e., someone who supported them through their cancer treatment) to complete a similar semi‐structured interview on cancer clinical trial experiences. Of the 26 caregivers contacted, 22 participated in the interview; however, one caregiver's interview was excluded due to technical audio‐recording problems. In total, 21 patient–caregiver dyads, or 42 total participants (i.e., 21 patients and 21 caregivers), were included in the current analysis. Among these dyads, about 75% of participants were enrolled in cancer clinical trials involving several weeks of radiation therapy, often in combination with chemotherapy. The remainder received chemotherapy‐based treatments that required travel to the treatment center a minimum of every 2–3 weeks.

Demographic information of patients and caregivers is shown in Table 1. Most caregivers were partners/spouses (*n* = 18, 85%), while two (10%) were other relatives (sister‐in‐law and niece) and one (5%) was an adult child. Additionally, 18 (85%) of patient–caregiver dyads lived in the same household during patient clinical trial participation, and the remaining three lived either within walking distance or up to a 20‐min car ride away.

**TABLE 1:** Demographics characteristics of patient and caregiver participants (*N* = 42).

Variable	Patient (*n* = 21)	Caregiver (*n* = 21)
Age, years (mean (SD))	73.71 (6.54)	69.95 (7.43)
Gender, *n* (%)		
Male	19 (90.5%)	1 (4.8%)
Female	2 (9.5%)	20 (95.2%)
Employment status, *n* (%)		
Retired	10 (47.6%)	8 (38.1%)
Not retired	11 (52.4%)	13 (61.9%)
Location,^a^ *n* (%)		
Rural	10 (47.6%)	10 (47.6%)
Urban	11 (52.4%)	11 (52.4%)
Caregiver type		
Spouse/partner	—	18 (85%)
Adult child	—	1 (5%)
Relative	—	2 (10%)
Caregiver living status		
Same household	—	18 (85%)
Other	—	3 (15%)

^a^ Location was determined by participant household zip codes and classified as urban or rural using the 2010 Rural–Urban Commuting Area (RUCA) codes.

#### 3.1.2 Perceived Caregiver Contributions

**FIGURE 1:**
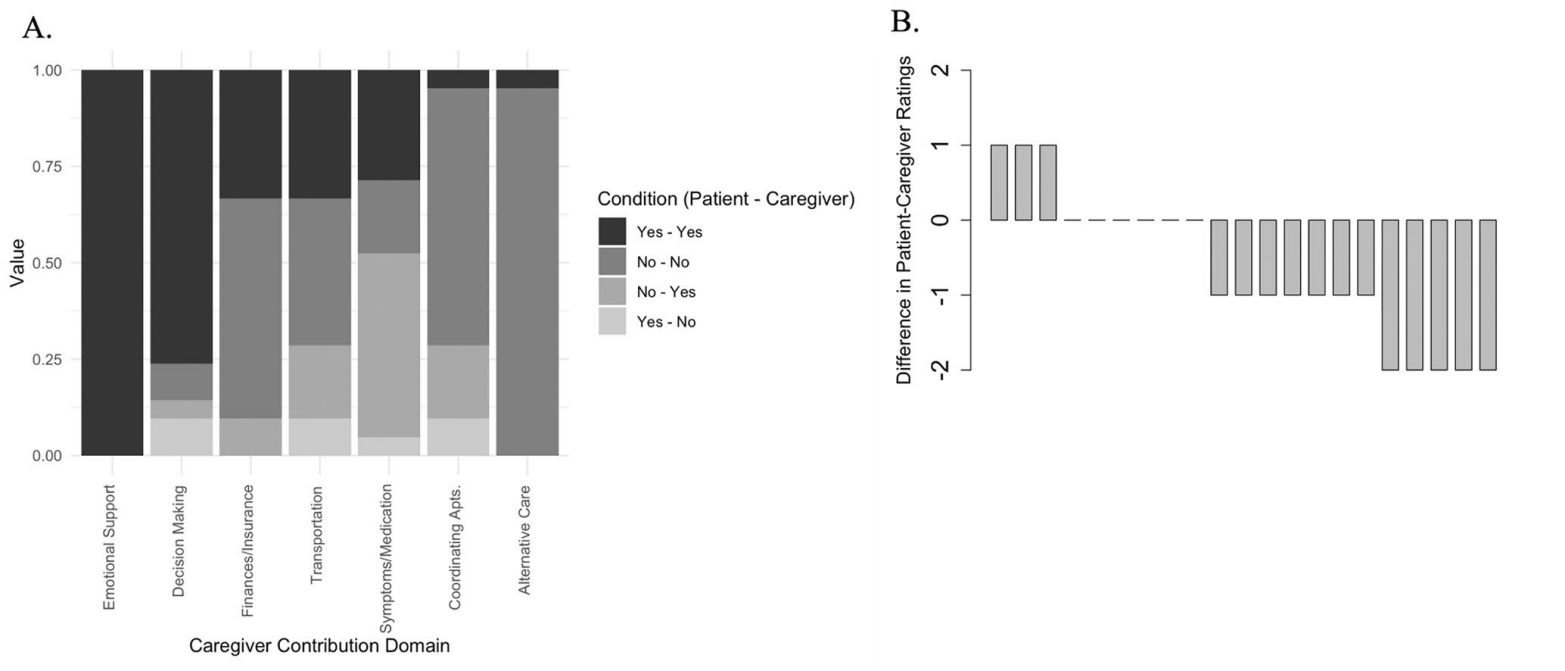
Differences in agreement and frequency of endorsed caregiver contributions by patient–caregiver dyad. (A) The proportion of agreement and disagreement in perceived caregiver contributions between patient–caregiver dyads (*N* = 21). The level of concordance/discordance is depicted across the seven caregiver contribution domains with four conditions (i.e., Yes–Yes, No–No, No–Yes, Yes–No). (B) The difference in total caregiver contribution ratings (i.e., sum of the seven domains) across each patient–caregiver dyad (*N* = 21). Negative values indicate caregivers reported a higher frequency of caregiver contributions than patients, and positive values indicate patients reported a higher frequency of caregiver contributions than caregivers. Values that fall on 0 indicate that the patient and caregiver rated total contributions equally. Among the 21 patient–caregiver dyads, 12 caregivers reported a higher frequency of caregiver contributions than patients, while six patient–caregiver dyads reported equal contributions and three patients reported a higher frequency than caregivers.

On average, patients reported caregivers assisted in 3.14 out of seven possible caregiver contribution domains (SD = 1.24), while caregivers reported assisting in an average of 3.18 (SD = 1.33) domains. As shown in Table 2, all patients and caregivers reported “yes” to emotional support. Treatment decision‐making (endorsed by 86% of patients and 81% of caregivers) was the next highest caregiver contribution domain, followed by symptoms/medication management (endorsed by 33% of patients and 76% of caregivers) and transportation (endorsed by 43% of patients and 52% of caregivers). There was a high level of agreement between patient–caregiver dyads regarding caregiver assistance, with over 50% of patient–caregiver dyads agreeing on whether caregivers provided help in the domains of emotional support, treatment decision‐making, finances/insurance, coordinating appointments, and arranging/providing alternative care (Table 3). In contrast, dyads often disagreed in the areas of transportation and symptom/medication management. However, the only significant discordance was found in the symptom/medication management domain, where 48% of patients reported “no” assistance while caregivers indicated “yes” (*χ* ^2^ (1, 21) = 5.82, *p* = 0.016). A visual comparison of the concordance and discordance in caregiver contributions is presented in Figure 1A, while Figure 1B shows the difference in total endorsed caregiver contributions (i.e., the sum of seven caregiver contribution domains) by each patient–caregiver dyad.

**TABLE 2:** Endorsed caregiver contributions by patient (*n* = 21) and caregiver (*n* = 21).

	Caregiver contribution domain, *n* (%)						
	Emotional support	Decision‐making	Symptoms/medication	Transportation	Finances/insurance	Coordinating appointments	Alternative care
Patient	21 (100%)	18 (86%)	7 (33%)	9 (43%)	7 (33%)	3 (14%)	1 (5%)
Caregiver	21 (100%)	17 (81%)	16 (76%)	11 (52%)	9 (43%)	5 (24%)	1 (5%)

**TABLE 3:** McNemar's chi‐square test results of patient–caregiver concordance and discordance.

Contribution domain	Caregiver—yes	Caregiver—no	*χ* ^2^	*p*
	*n*	*n*		
Symptoms/medication				
Patient—yes	6	1	5.82	**0.016**
Patient—no	10	4		
Decision‐making				
Patient—yes	16	2	0.00	1.00
Patient—no	1	2		
Transportation				
Patient—yes	7	2	0.17	0.683
Patient—no	4	8		
Finances				
Patient—yes	7	0	0.50	0.480
Patient—no	2	12		
Coordination				
Patient—yes	1	2	0.17	0.683
Patient—no	4	14		
Emotional support				
Patient—yes	21	0	—	—
Patient—no	0	0		
Alternative care				
Patient—yes	1	0	—	—
Patient—no	0	20		

*Note:* McNemar's chi‐square tests were performed to compare the relative concordance/discordance of perceived caregiver contributions between patient–caregiver dyads (*N* = 21) across the seven caregiver contribution domains. The frequency of agreement and disagreement between dyads is represented by four conditions: (1) both the patient and caregiver reported a contribution (i.e., Patient‐Yes, Caregiver‐Yes), (2) the patient acknowledged a contribution while the caregiver did not (i.e., Patient‐Yes, Caregiver‐No), (3) the caregiver acknowledged a contribution while the patient did not (i.e., Patient‐No, Caregiver‐Yes), and (4) neither the patient nor caregiver reported a contribution (i.e., Patient‐No, Caregiver‐No). Degrees of freedom is 1 for all tests. Emotional support and alternative care domains were excluded from analyses due to bin sizes of 0. Bolding indicates significant *p* < 0.05.

### 3.2 Qualitative Results

Qualitative analysis of interview data identified patterns within the expected roles for caregivers during patient clinical trial participation. Three major themes were identified: patient independence, invisible support, and accepted forms of support.

#### 3.2.1 Patient Independence

Patients spoke to both exercising and valuing their independence during cancer clinical trial participation. Many patients described themselves as not needing assistance from others during their clinical trial participation, despite caregivers being present if they needed help.

I'm pretty self‐sufficient so… If I needed anything, he would have been right there with me, but I really didn't need him… (Patient 2, Rural)

Caregivers confirmed the theme of patient independence, describing patients as “doing things [himself],” “very capable,” and not wanting to be “babied” or “hovered over.”

Like I said she's very independent and she's a very smart woman. And she was able to do a lot of the research. She likes to manage herself. (Caregiver 6, Urban)

However, caregivers also acknowledged that cancer had an important impact on them, even if they felt patients were trying to protect them. One caregiver described the patient as wanting to carry the burden:

As soon as he could go to his radiation appointments on his own, he didn't want me to go with him anymore. I think because he didn't want to make me have to do any extra. But we were both experiencing it, only I think he wanted to carry the burden more himself because he didn't want to make me. (Caregiver 12, Urban)

#### 3.2.2 Invisible Support

Caregivers sometimes reported providing support that may have been less noticed by patients, echoing quantitative results showing that, within most patient–caregiver dyads, caregivers reported higher overall caregiver involvement than patients. While most patients reported coordinating appointments and managing their treatment schedules independently, several caregivers reported some involvement in this activity, primarily around helping to keep track of treatment schedules and ensuring patients did not miss any appointments.

I made sure that things were on the calendar, you know? And then I would ask him if he had dates that we needed to put down on the calendar. (Caregiver 9, Rural)

Additionally, several caregivers described not asking too much of the patient during the clinical trial and proactively handling more household‐related tasks than before the trial began.

I mean I could see the impact. So I just backed off—and not only backed off from expecting him to do things, realized that I had to step in and do things that he used to do. (Caregiver 1, Urban)

However, these contributions were not always mentioned by patients, or patients minimized caregivers' involvement.

She'd take over some of the—a few of things when I was really tired after the radiation treatment … It was really minor. We just changed duties a little bit at home. That's all. (Patient 1, Urban)

Several caregivers also described providing coaching to the patient behind the scenes; for example, encouraging patients to call the hospital or insurance company with concerns.

Regularly saying—coaching like ‘you need to call the hospital, you need to get the details of the bill, these dates are not correct,’ you know? (Caregiver 20, Urban)

Medication/symptom management was one domain in which patient–caregiver dyads showed significant disagreement in their perceptions of caregiver involvement, with caregivers tending to endorse a contribution while patients did not. In terms of medication management, caregivers reported assisting with prescription refills and aiding in medication adherence. For example, one caregiver even implemented a medication tracking system to help the patient keep track of their daily intake.

I got him a clipboard because he was keeping the paper wherever and losing it. So, I got him a clipboard so he could keep the paper on the clipboard. It was a fluorescent orange clipboard, so no losing that. (Caregiver 12, Urban)

Regarding symptom management, most caregivers reported assisting with patient side effects by helping patients be more comfortable (e.g., keeping the house cool), accommodating diets, aiding in bathroom‐related side effects, and ensuring patients could rest when fatigued.

#### 3.2.3 Accepted Forms of Support

While patients reported wanting to maintain independence, a few types of caregiver support emerged as “acceptable” for patients. These largely included offering emotional support, attending appointments together, and being informed. Emotional support was the only caregiver contribution domain endorsed by every patient and caregiver and was widely accepted.

I mean I don't feel like I needed a lot of support other than talking about the emotional side of having cancer. I didn't need a lot of support with the treatments. They weren't debilitating or anything, so I didn't need a lot of hands‐on care. (Patient 18, Rural)

Emotional support took many forms but primarily included caregivers listening and talking to the patient about their treatment experience, being a “sounding board,” checking in, as well as providing sympathy, encouragement, and distraction.

Letting him vent when he wasn't feeling well. I made him baked goods to try to perk him up a little. Just spending time with him and keeping him kind of occupied. Those types of things. (Caregiver 16, Urban)

Additionally, receiving and providing emotional support appeared to positively influence both the patient and caregiver experience throughout clinical trial participation.

In just being able to come back and have somebody ask ‘well, how did it go?’ Or ‘you're a little bit later today, did everything go ok?’ Just discussion is very helpful, you know? (Patient 6, Urban)

I feel valued, I will say that. Multiple times a day, he will say ‘I don't know how I would have been able to go through this by myself’. So, I just think the emotional support is the most important. (Caregiver 15, Urban)

In addition to providing emotional support, most caregivers and patients reported attending at least the initial clinical trial appointment together, during which clinical trial participation was first discussed with the patient. Several caregivers and patients reported attending multiple appointments together, especially during the beginning of clinical trial participation, and some caregiver‐patient dyads reported attending every appointment together throughout the duration of the patient's clinical trial.

I just told him I was going with him. He has difficulty hearing, even with his hearing aids. So, I felt as for moral support as well as to be the second set of ears, it was necessary for me to go. And I wanted to support him. (Caregiver 12, Urban)

Although attending appointments together was more common for urban patient–caregiver dyads than rural dyads, virtually all patients and caregivers mentioned it was helpful to have “a second pair of ears in the room” and to ease worries around memory/information bias and accurate record keeping.

I went to his doctor's appointments with him and took notes and just made sure we both had a good understanding of what the doctors were saying. (Caregiver 8, Rural)

Additionally, having knowledge of the clinical trial process was reported by caregivers to be helpful in terms of what questions to ask and when, as well as tempering expectations, understanding side effects, and advocating for patient needs.

I think just having heard everything at the same time, it made me more understanding when he said ‘oh, I got a hot flash’ or he is exhausted in the afternoons. Not that I would have ever said ‘oh my god, you're napping again’ or something like that [laughs]. It helped me understand the journey he was going to go through and is going through. (Caregiver 15, Urban)

Beyond getting information at appointments, many caregivers, especially rural caregivers, described either reading pamphlets and other handouts sent home with the patient or researching cancer information online. Both caregivers and patients described such informational readings as helpful for caregivers' understanding of patient experiences.

I read up on what was going on, probably not in as much details as he did. But I felt like I was tuned into it and with him the whole time. (Caregiver 19, Rural)

I would give her stuff to read. The radiation oncologist would give me little pamphlets and things to read, which were very well done. And I'd give them to her, and she read every word of them, so she knew what was going on. (Patient 19, Rural)

Patients and caregivers also spoke to how the caregiver being informed and obtaining knowledge about the clinical trial and cancer diagnosis/treatment process could be beneficial in providing emotional support.

I think it helped him to know that I was up to date with what he was going through. (Caregiver 15, Urban)

She understood what I was going through and just kept me centered. (Patient 3, Urban)

## 4 Discussion

The current study investigated both patient and caregiver perceptions of caregiver contributions during cancer patient clinical trial experiences. As with the broader cancer caregiving population, caregivers of patients on clinical trials reported assisting patients in numerous ways, including providing emotional support, aiding in treatment‐related decision‐making, mitigating patient side effects, and assisting in medication management.

While patient–caregiver dyads showed significant disagreement in the symptoms/medication management domain, both groups were typically in agreement and reported similar levels of caregiver involvement across the other six caregiver domains assessed. However, qualitative results revealed that caregivers perceived a higher level of involvement in care than patients reported.

Caregivers' initial responses to close‐ended questions assessing the seven caregiver contribution domains were often expanded upon in open‐ended responses, revealing additional ways they helped within specific domains. For example, while most patients and caregivers reported no caregiver assistance in coordinating appointments, open‐ended caregiver responses showed that caregivers assisted patients in managing calendars and reinforcing treatment schedules. These discrepancies might have occurred from the intertwined nature of caregiving and familial roles. Prior research indicates that caregivers, especially spouses/partners, often see their roles as familial duties, not explicitly as caregiving tasks [25, 26, 27]. Our study sample, primarily consisting of spouses/partners, likely reflected this dynamic. Thus, caregivers may not have perceived their caregiving contributions as distinct from their relationship‐based roles or marital duties, which influenced quantitative results.

Additionally, there was a strong theme of independence among patients in our sample, with some patients attempting to uphold their independence to lessen the burden on their caregiver during the trials. This desire for independence, which was recognized by caregivers, may have pushed caregivers to support patients discreetly, allowing patients to overlook or minimize their caregivers' contributions while caregivers smoothed out the patient clinical trial experience from behind the scenes. Our study highlights that while patients in cancer clinical trials may appear to navigate the process independently, caregivers are providing crucial support behind the scenes. This lack of recognition of caregiver support is consistent with prior research that finds caregivers often feel invisible due to factors such as being excluded from care planning conversations, dismissed in clinical encounters, and minimizing their own needs [8, 28, 29, 30].

Interestingly, our qualitative results did not reveal any major differences between rural and urban patient–caregiver dyads in terms of caregiver contributions. One potential explanation for this result may be our sample's enrollment in cancer clinical trials, which have been shown to improve access to quality care among rural cancer patients and reduce health disparities between rural and urban cancer patients [9]. However, a few differences between rural and urban caregivers emerged from the qualitative analysis. Namely, urban caregivers attended more appointments with patients than rural caregivers, and rural caregivers spoke more about being informed than urban caregivers. For rural caregivers, being informed may have been a means to feel more involved in care and compensate for not attending appointments with patients due to geographical and other resource barriers.

### 4.1 Study Limitations

This study had significant strengths, including a mixed‐methods approach and dyadic analysis of both patient and caregiver reports. However, there were important study limitations. Most notably, our sample was predominantly non‐Hispanic White and included mainly older male patients and female caregivers. Racial/ethnic and age variables play an important role in interfacing with the healthcare system, influencing treatment experiences/outcomes [31, 32] and the caregiving experience [33, 34]. Our results do not address how these factors, particularly among minoritized individuals and younger patients, impact caregiver contributions during patient clinical trial experiences. Additionally, all patients were currently enrolled in a cancer clinical trial upon participation in the current study. Thus, we were unable to systematically compare how caregiving contributions may differ for patients enrolled or not in a clinical trial. Notably, prior research has identified multiple barriers to cancer clinical trial recruitment and participation among historically marginalized racial and ethnic groups, including limited awareness of clinical trials, eligibility restrictions, mistrust, financial costs, and transportation challenges [35]. These barriers, among others, contribute to poor representation in cancer clinical trials, further exacerbating racial and ethnic health inequities [36]. Future research is warranted to explore how factors such as race/ethnicity, age, and clinical trial status influence the caregiving experience during cancer treatment. Lastly, our statistical findings should be interpreted with caution due to the relatively small sample size, which limited our statistical power. The sample size also prevented us from conducting a robust quantitative analysis of differences between rural and urban participants. Future studies with larger sample sizes are encouraged to explore these differences, as they are likely to yield valuable insights.

### 4.2 Clinical Implications

Caregivers are very involved throughout the patient clinical trial experience, seeking information and providing support, which can be particularly challenging with independent patients. Patients' desire for independence, coupled with caregivers minimizing their role, complicates understanding how caregivers support patients at home and how best to assist them. Our findings highlight the need for clinical trials to integrate and support caregivers from the outset. Clinical staff should enlist caregivers upon initial enrollment in clinical trials, identifying them in patient medical records to facilitate integration, and offer support through education and tailored resources on cancer treatment and caregiving. Providing caregivers with information on their vital role in cancer treatment can help increase their visibility as key care team members, ease the transition from a relationship‐based role to an active caregiving one, and subsequently encourage caregivers to use support resources early. Studies have shown that supporting caregivers through early interventions like psychoeducation and skills development (e.g., coping, problem‐solving) positively affects caregiver psychological well‐being and, in turn, patient quality of life [7, 37, 38].

Given the benefit of caregivers being informed during patient clinical trial participation, clinical staff should consider routinely providing take‐home handouts and knowledge resources tailored to caregivers, especially in rural patient populations, to empower caregivers with information and consequently enhance their ability to support patients. With patient consent, providers should routinely invite caregivers to appointments, especially informational and decision‐based meetings, and offer telemedicine options for rural caregivers to overcome attendance barriers. Overall, these strategies may increase the visibility of caregiver contributions and enhance support for both caregivers and patients during cancer clinical trials.

## 5 Conclusions

Our study sheds light on the multifaceted role of caregivers in supporting cancer patients enrolled in clinical trials. While quantitative results showed similarities in reported caregiver contributions between patients and their caregivers, as well as general agreement within dyads, qualitative insights revealed the often unseen and underestimated ways in which caregivers provided support. Cancer clinical trial retention and outcomes are likely to benefit from an increased focus not just on the patient, but on the family system. Interventions and strategies aimed at supporting caregivers, especially rural caregivers with unique, often unmet needs, are needed within clinical trials to better recognize and assist caregivers. Future clinical trials should adopt a family‐centered approach to incorporate this broader perspective.

## Author Contributions

**Janine Cerutti:** data curation (supporting), formal analysis (lead), methodology (equal), writing – original draft (lead), writing – review and editing (equal). **Maria C. Lent:** conceptualization (equal), data curation (lead), methodology (equal), project administration (lead), writing – review and editing (equal). **Randall F. Holcombe:** conceptualization (equal), formal analysis (supporting), funding acquisition (lead), methodology (equal), writing – review and editing (equal). **Maija Reblin:** conceptualization (equal), formal analysis (supporting), methodology (equal), writing – review and editing (equal).

## Conflicts of Interest

The authors declare no conflicts of interest.

## Data Availability

The data are not publicly available due to privacy or ethical restrictions.
